# Expression of Cyclin D1 gene in ovarian cancer and effect of silencing its expression on ovarian cancer cells based on the Oncomine database

**DOI:** 10.1080/21655979.2021.2000225

**Published:** 2021-11-22

**Authors:** Li-Li Quan, Jin-Yang Liu, Li-Xia Qu, Hui La, Hai-Li Wang, Xiao-Xia Chen, Na Wang, Zhen-Zhi Wei

**Affiliations:** a Department of Gynecology, Sanmenxia Central Hospital of Henan University of Science and Technology, Sanmenxia, Henan Province, China; b Department of Anesthesiology, Sanmenxia Central Hospital of Henan University of Science and Technology, Sanmenxia, Henan Province, China

**Keywords:** Oncomine, Cyclin D1, ovarian cancer, silencing

## Abstract

We aimed to analyze the expression of Cyclin D1 (CCND1) gene in ovarian cancer and the influence of silencing its expression on ovarian cancer cells based on the Oncomine database. The expression of CCND1 gene in ovarian cancer was analyzed by utilizing the relevant information in different tumors and Oncomine database. The correlation between CCDN1 expression level and prognosis of ovarian cancer was analyzed by the online database Kaplan-Meier (kmplot.com). The expression of CCND1 gene in ovarian cancer and the effect of silencing its expression on cancer cells were analyzed by cell experiments. After mining and comprehensively analyzing 7 studies on the differential expression of CCND1 gene in ovarian cancer tissue and normal ovarian tissue included in the Oncomine database, it was found that the median value of CCND1 gene ranked 218.0 (P = 8.03 × 10^−6^) among all differentially expressed genes, suggesting that CCND1 gene expression in ovarian cancer tissue was higher than that in normal ovarian tissue. Adib Ovarian, Bonome Ovarian and Hendrix Ovarian microarrays revealed that the expression of CCND1 gene in ovarian cancer tissue was significantly higher than that in normal ovarian tissue (P < 0.05). Kaplan-Meier Plotter database showed that the overall survival and progression-free survival of ovarian cancer patients with high CCND1 expression were significantly shorter than those of patients with low CCND1 expression (P < 0.05). The expression levels of CCND1 gene in normal ovarian epithelial cells and SKOV3 ovarian cancer cells were detected by RT-PCR. The expression of CCND1 gene was significantly higher in SKOV3 group than that in control group (P < 0.01). Flow cytometry revealed that the percentage of cells in G_0_/G_1_ phase was significantly higher, while that in S phase was lower in SKOV3 + siCCND1 group than the values of SKOV3 and SKOV3 + siNC groups (P < 0.05). The apoptosis rate of ovarian cancer cells was significantly higher in SKOV3 + siCCND1 group than those of SKOV3 and SKOV3 + siNC groups (P < 0.01). CCND1 gene is highly expressed in ovarian cancer tissue and related to prognosis. Preoperative evaluation of CCND1 gene expression in ovarian cancer patients may benefit the assessment of risk and prognosis.

## Introduction

Ovarian cancer is one of the most common gynecological malignancies with the highest mortality rate in the female reproductive system globally. According to the data of the National Cancer Center of China, there are over 40,000 new cases of ovarian cancer and more than 10,000 deaths annually in China [1]. However, due to recurrence, delayed diagnosis and chemotherapy resistance, the 5-year survival rate of patients with advanced ovarian cancer is about 45–50% [2]. Similar to solid tumors, the occurrence and development of ovarian cancer involves a variety of factors and genes, which is a complicated molecular process. Hence, identifying the key genes associated with the occurrence and development of ovarian cancer and determining the effective targets of gene therapy are of profound significance for early diagnosis and treatment and elevation of survival rate. With the rapid development of bioinformatics and high-throughput sequencing, tumor-related databases have become a useful tool for mining tumor-related genes. Oncomine (www.oncomine.org), the biggest oncogene microarray database worldwide, is a data mining platform for exploring tumor-related genes, which integrates The Cancer Genome Atlas, Gene Expression Omnibus and the deoxyribonucleic acid-sequencing (DNA-seq) and ribonucleic acid (RNA) data from published literatures. Cyclin D1 (CCND1) is one of the cell cycle regulators modulating the occurrence and development of malignant tumors, which is typified by overexpression. CCND1 gene is abnormally expressed in various tumors, such as glioma [3], cervical cancer [4], and gastric cancer [5].

Until now, there are few studies on the expression of CCND1 gene in ovarian cancer, and its role and function remain unclear. Thereby motivated, we herein aimed to investigate the expression and mechanism of CCND1 gene in ovarian cancer in order to clarify the mechanism for the occurrence and development of ovarian cancer at the level of molecular biology.

## Materials and methods

### Data collection

The Oncomine database is an analysis platform collecting gene microarray data. In this study, ovarian cancer-related data were screened from this database under the following conditions: (1) Cancer Type: ovarian cancer, (2) Gene: CCND1, (3) Analysis Type: Cancer *vs*. Normal Analysis, (4) Data Type: DNA or messenger RNA (mRNA), (5) Critical value: fold change >2, P-value <10^−4^, and gene rank = top 10%.

### Materials

Human ovarian cancer cell line SKOV3 (human ovarian adenocarcinoma cells) and normal ovarian epithelial cells were purchased from the Cell Center of Peking Union Medical College. Rabbit anti-CCND1 was bought from Cell Signaling Technology (USA). CCND1 small interfering RNA (siRNA) and negative control si-RNA (N-siRNA) were sourced from Sigma (USA). Lipofectamine was bought from Invitrogen (USA), Dulbecco’s Modified Eagle Medium (DMEM)-1640 was provided by Gibco (USA), and TRIzol kit was purchased from Thermo Fisher Scientific (USA).

### Cell culture

Normal ovarian epithelial cells and SKOV3 cells were cultured as follows: (1) Cell resuscitation: After the thermostatic water bath box was preheated to 37°C, the cells frozen in liquid nitrogen were taken out and immediately put into the water bath box. Later, the cell suspension was aspirated into a sterile centrifuge tube on a superclean bench, and added with an appropriate amount of DMEM-1640, followed by centrifugation at 1,000 r/min for 5 min. Then the supernatant was discarded. After the culture medium was added, the cells were transferred into a culture flask, and the culture medium was replaced every 2–3 d. (2) Cell passage: The cells were passaged when they reached about 85% confluence, and washed twice with preheated D-Hanks. Then trypsin-ethylenediaminetetraacetic acid (EDTA) solution (0.25% trypsin and 0.02% EDTA) was added for 2 min of digestion. Afterward, the cells were observed under a microscope. When the cells became round and retraction of cell protuberance was observed, the culture medium was added to terminate digestion. After centrifugation at 1,000 r/min for 5 min, the supernatant was discarded. Subsequently, the culture medium was added and mixed evenly by blowing and suction, followed by subpackage into culture flasks. (3) Cell cryopreservation: After the cells were digested by trypsin-EDTA solution, the culture medium was added, followed by centrifugation at 1,000 r/min for 5 min. The, the supernatant was discarded, and the cells were added with aseptic cryopreservation solution and subpackaged. After being kept at 4°C for 30 min, −20°C for 2 h, and −80°C for 24 h, they were preserved at −150°C. (4) Cell culture: The cells were cultured in an incubator with 5% CO_2_ at 37°C, and the cell morphology was observed using a phase contrast microscope.

### Detection of CCND1 expressions in ovarian epithelial and SKOV3 cells by RT-PCR

Total RNA was extracted according to the TRIzol method, and complementary DNA was obtained by reverse transcription, 2 μL of which was used for PCR. CCND1 primer sequences: upstream primer: 5ʹ-CGGTCTTGAAGGGTTCCTTTC-3ʹ, downstream primer: 5ʹ-CCAAGTCCACTGTGGTGTTTAAG-3ʹ. β-Actin primer sequences: upstream primer: 5ʹ-TGAATCAACGCAATGTGGGAA-3ʹ, downstream primer: 5ʹ-CAGTGGAAGTGGCAAGGT-3ʹ. The total reaction volume was 20 μL, and the amplification conditions were as follows: pre-denaturation: 95°C for 30 s, and amplification: 40 cycles of 95°C for 5 s, 57–59°C for 30 s, and 72°C for 30 s. β-Actin was used as the internal reference for correction. The relative expression of gene was analyzed by the 2^−∆∆Ct^ method, which was repeated 3 times [6].

### Transfection of SKOV3 cells with plasmids

(1) SKOV3 cells were transferred to a 6-well cell culture plate at 1 × 10^5^/mL, and cultured in an incubator with 5% CO_2_ at 37°C for 1 d. When 80% of the culture plate was covered by cells, the culture medium was discarded, and replaced by DMEM-1640 for culture overnight. (2) 250 μL of serum-free DMEM-1640 was added to 10 μL of Lipofectamine 2000. After the wall of centrifuge tube was gently flicked, the cells were incubated at room temperature for 5 min. (3) 250 μL of serum-free DMEM-1640 was added in 3 centrifuge tubes, respectively. The screened si-RNA with silenced CCND1 gene was used (CCND1-siRNA sequence: 5ʹ-UTUGGUUTTTCUGTCCCGUUUTCGC-3ʹ and N-siRNA sequence: 5ʹ-CTCUCCCUGUCCCUGUUGGGCUTCT-3ʹ). Two of the tubes were added with 4 μg of N-siRNA plasmid (for transfection of negative siRNA) and 4 μg of CCND1-siRNA plasmid (for transfection of CCND1-siRNA), respectively, and the wall of the centrifuge tubes was gently flicked. (4) Lipofectamine 2000 diluent was added with plasmid, followed by incubation at room temperature for 20 min to make the liposome encapsulate the plasmid. (5) The mixture of DNA-liposome was added to the 6-well cell culture plate, mixed evenly and incubated in an incubator with 5% CO_2_ at 37°C for 4–6 h. After the culture medium was discarded and replaced with normal culture medium, the cells were cultured for another 48 h. Subsequently, the morphology of the cells after transient transfection was observed under an inverted microscope. The stable cell lines were screened by puromycin (4 μg/mL) and then digested by EDTA-trypsin. Later, the cells were passaged at a ratio of 1:4 and partly cryopreserved [7].

### Detection of cell cycle distribution

Cell cycle detection was conducted according to the instructions of cell cycle kit (BD Cycletest™ Plus DNA Reagent Kit). After digestion with EDTA-free trypsin, the cells were centrifuged and collected. Later, they were added with 70% anhydrous ethanol and kept in dark at 4°C overnight. After ethanol was washed away by centrifugation, propidium iodide (PI) staining solution was added in dark for reaction for 30 min. Finally, the fluorescence intensity at Ex = 488 nm was detected by flow cytometry, and the percentages of cells were analyzed. Three groups were set, i.e. SKOV3 group, SKOV3 + siNC group and SKOV3 + siCCND1 group [8].

### Detection of cell apoptosis

Apoptosis detection was performed according to the instructions of apoptosis kit (BD Pharmingen™ Annexin V: PE Apoptosis Detection Kit I). After digestion with EDTA-free trypsin, the cells were centrifuged and collected. Later, the cells were resuspended with binding buffer and incubated with Annexin V-FITC and PI staining solution successively. Finally, the fluorescence intensity at Ex = 488 nm and Em = 530 nm was detected by flow cytometry, and apoptosis rate was analyzed. The experimental groups were the same as those in cell cycle detection. Each treatment was repeated 3 times [9].

### Statistical analysis

GraphPad Prism 5.01 software was utilized for plotting, and SPSS 19.0 software was used for data entry and statistical analysis. One-way analysis of variance was employed for comparisons among groups, and *t*-test was used for comparisons between groups. The relationship between CCDN1 expression level and prognosis of ovarian cancer patients was analyzed using the online database Kaplan-Meier (kmplot.com). GeneCards (http://www.genecards.org) was utilized to search the relevant information and related proteins of CCND1 gene, and the network diagram of related proteins of CCND1 gene was constructed by STRING (https://string-db.org/cgi/input.pl). P < 0.05 suggested that a difference was statistically significant.

## Results

### Expressions of CCND1 gene in different types of tumors

CCND1 gene amplification occurs in 20% of human ovarian cancer and squamous cell carcinoma [10]. In normal cells, CCND1 protein promotes the transition from G1 phase to S phase, which is related to cell proliferation. Therefore, CCND1 may be closely associated with the prognosis of ovarian cancer patients.

**Figure 1.**
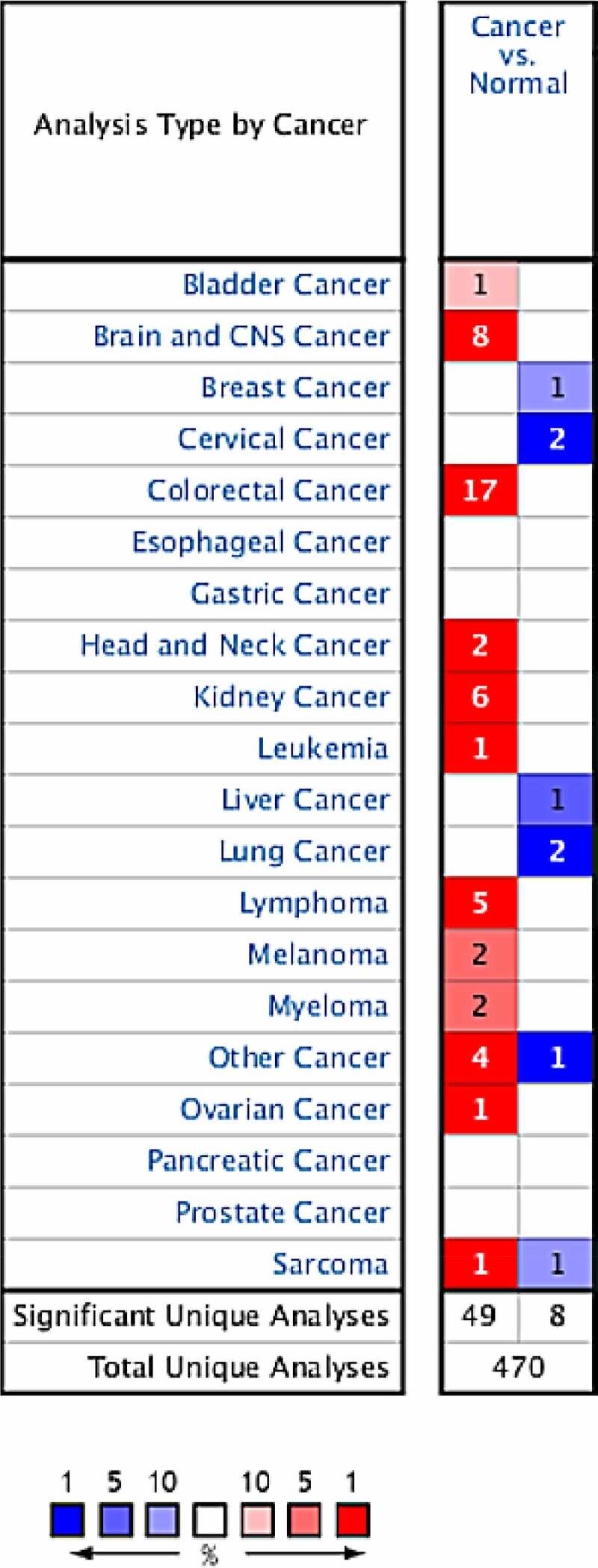
Expressions of CCND1 gene in different types of tumors in Oncomine database (blue: low expression, red: high expression, comparison within the same line)

After retrieving and analyzing the Oncomine database, 470 correlation analysis results on CCND1 genes were included. Among them, 49 cases of high expression of CCND1 gene and 8 cases of low expression of CCND1 gene with significant differences were selected for further analysis as follows: 1 case of high expression and 0 case of low expression in bladder cancer, 8 cases of high expression and 1 case of low expression in brain and central nervous system tumors, 0 case of high expression and 1 case of low expression in breast cancer, 0 case of high expression and 2 cases of low expression in cervical cancer, 17 cases of high expression and 0 case of low expression in colorectal cancer, 2 cases of high expression and 0 case of low expression in head and neck tumors, 6 cases of high expression and 0 case of low expression in renal carcinoma, 1 case of high expression and 0 case of low expression in leukemia, 0 case of high expression and 1 case of low expression in liver cancer, 0 case of high expression and 2 cases of low expression in lung cancer, 5 cases of high expression and 0 case of low expression in lymphoma, 2 cases of high expression and 0 case of low expression in melanoma, 2 cases of high expression and 0 case of low expression in myeloma, 4 cases of high expression and 1 case of low expression in other cancers, 1 case of high expression and 1 case of low expression in sarcoma, and 1 case of high expression and 0 case of low expression in ovarian cancer (Figure 1).

### Expression of CCND1 gene in ovarian cancer

**Figure 2.**
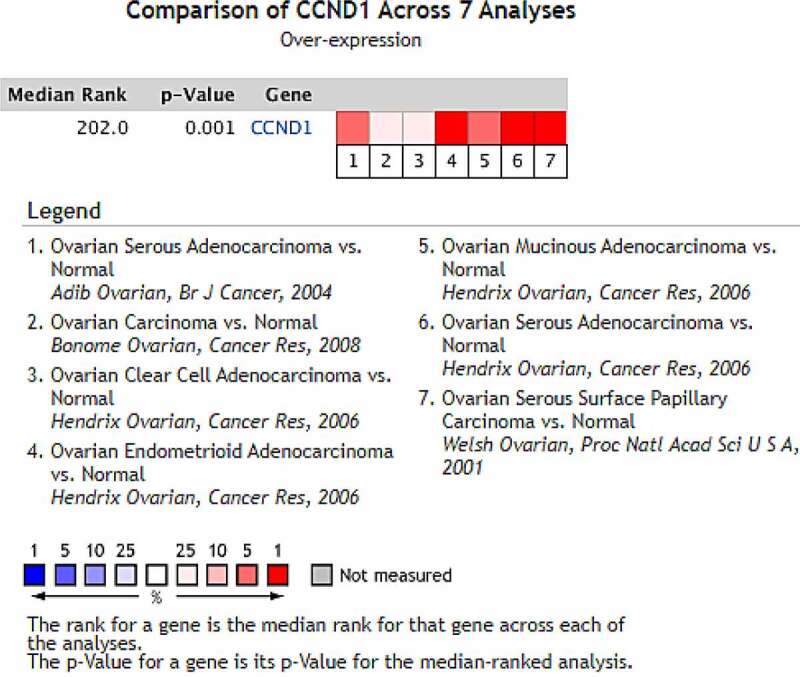
Expression of CCND1 gene in ovarian cancer in Oncomine database

After mining and comprehensively analyzing 7 studies on the differential expression of CCND1 gene in ovarian cancer and normal ovarian tissue included in the Oncomine database (the articles were published in the *British Journal of Cancer, Cancer Research* and *Proceedings of the National Academy of Sciences of USA*, respectively), it was found that compared with control group (normal ovarian tissue), the median value of CCND1 gene ranked 218.0 (P = 8.03 × 10^−6^) among all differentially expressed genes, indicating that the expression of CCND1 gene in ovarian cancer tissue was higher than that in normal ovarian tissue (Figure 2).

### Expressions of CCND1 gene in different ovarian cancer research microarrays

**Figure 3.**
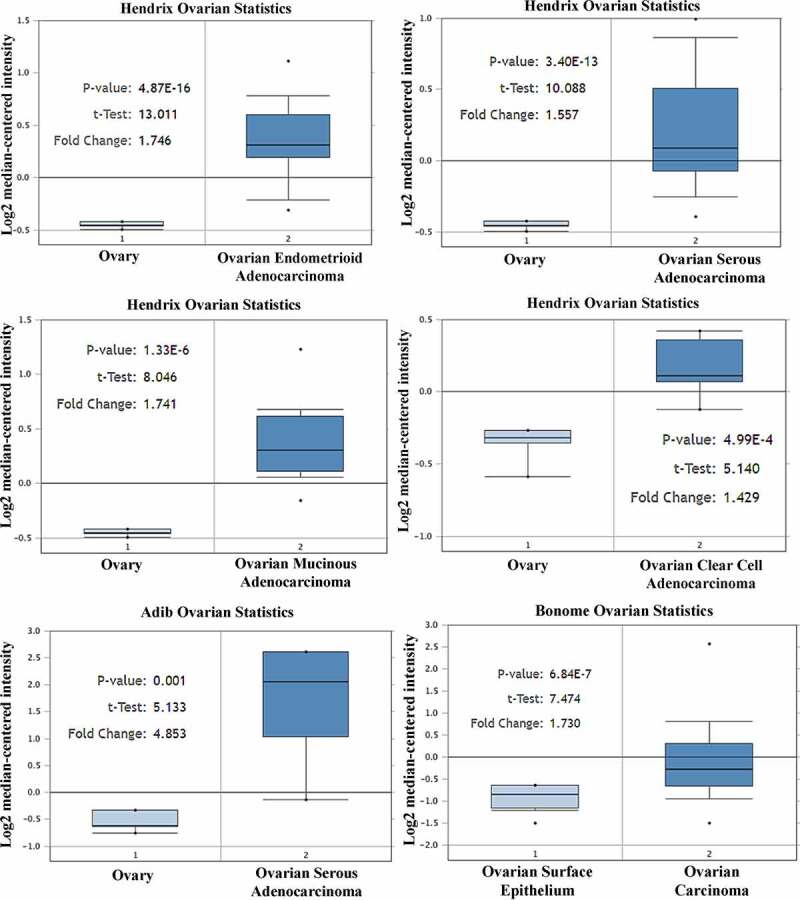
Expressions of CCND1 gene in different ovarian cancer research microarrays

By studying the Adib Ovarian, Bonome Ovarian and Hendrix Ovarian microarrays, it was found that the expression of Cyclin D1 gene in ovarian cancer tissue was significantly higher than that in normal ovarian tissue (P < 0.05) (Figure 3). Therefore, there was significant difference in the expression of CCND1 gene between ovarian cancer and normal ovarian tissue, which was highly expressed in ovarian cancer tissue.

### Effect of CCND1 gene on survival rate of ovarian cancer patients

CCND1 is overexpressed in ovarian cancer tissues. The expression of CCND1 in advanced ovarian cancer tissues is higher than that in early ones, and the prognosis of patients with high CCND1 expression is poor [11].

**Figure 4.**
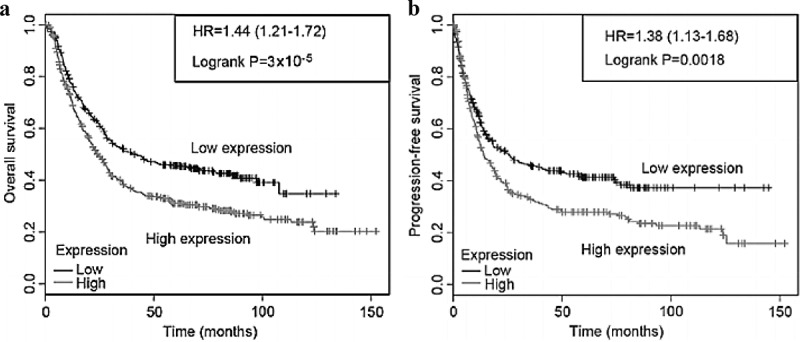
Effect of CCND1 gene on survival rate of ovarian cancer patients

The survival of ovarian cancer patients was analyzed based on the Kaplan-Meier Plotter database, and the correlations between CCND1 and overall survival (OS) and progression-free survival (PFS) of ovarian cancer patients were retrieved online. Thus, OS and PFS of ovarian cancer patients with high CCND1 expression were significantly shorter than those of patients with low CCND1 expression (P < 0.05) (Figure 4).

### Expressions of CCND1 gene in normal ovarian epithelial cells and SKOV3 ovarian cancer cells

**Figure 5.**
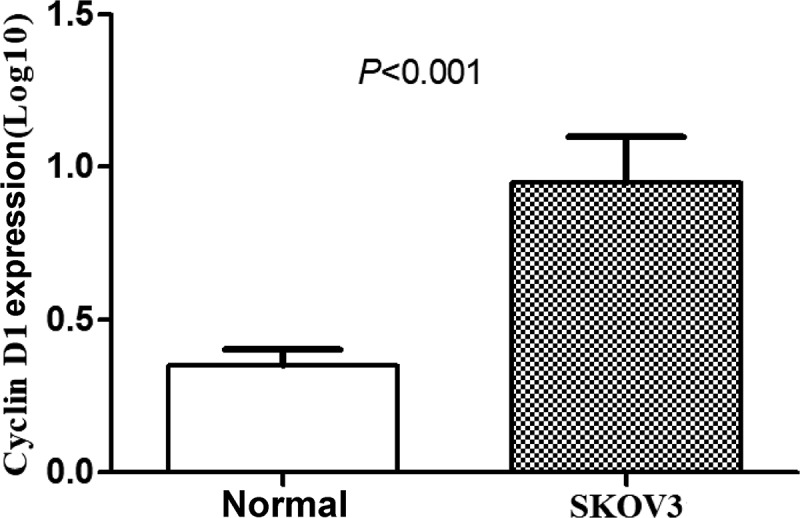
Expressions of CCND1 gene in normal ovarian epithelial cells and SKOV3 ovarian cancer cells

The expressions of CCND1 gene in normal ovarian epithelial cells and SKOV3 ovarian cancer cells were detected by RT-PCR, and the relative expression of CCND1 gene was calculated. The expression of CCND1 gene in SKOV3 group was significantly higher than that in normal group (P < 0.01) (Figure 5).

### Effect of silencing CCND1 gene on ovarian cancer cell cycle

**Figure 6.**
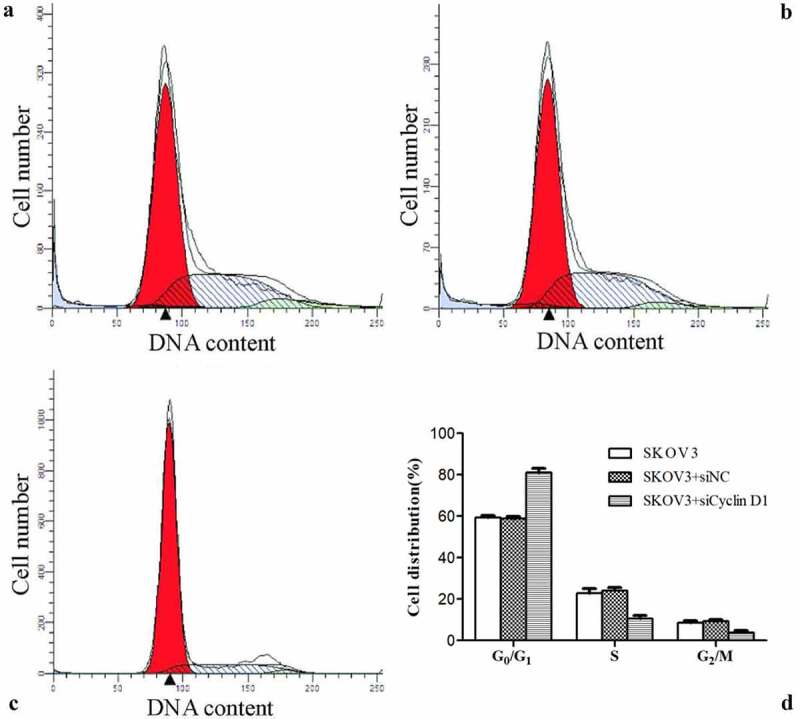
Effect of silencing CCND1 gene on ovarian cancer cell cycle. A/B/C: SKOV3, SKOV3 + siNC and SKOV3 + siCCND1. D: Comparison of cell cycle distribution among the three groups (*P < 0.01 *vs*. SKOV3 group, ^#^P < 0.05 *vs*. SKOV3 + siNC group)

The cell cycle distribution was detected by flow cytometry. The percentage of cells in G_0_/G_1_ phase was significantly higher, while that in S phase was lower in SKOV3 + siCCND1 group than the values of SKOV3 and SKOV3 + siNC groups (P < 0.05). However, the percentages of cells in G_0_/G_1_ phase and S phase were not significantly different between SKOV3 and SKOV3 + siNC groups. Accordingly, compared with SKOV3 and SKOV3 + siNC cells, SKOV3 + siCCND1 cells had inhibited transition of cell cycle from G_0_/G_1_ phase to S phase (Figure 6).

### Effect of silencing CCND1 gene on apoptosis of ovarian cancer cells

As a cell cycle-regulatory protein, CCND1 can obviously inhibit the proliferation, infiltration and metastasis of lung adenocarcinoma A549 cells after down-regulation of its expression [12]. Therefore, the effect of silencing CCND1 gene on the apoptosis of ovarian cancer cells was evaluated.

**Figure 7.**
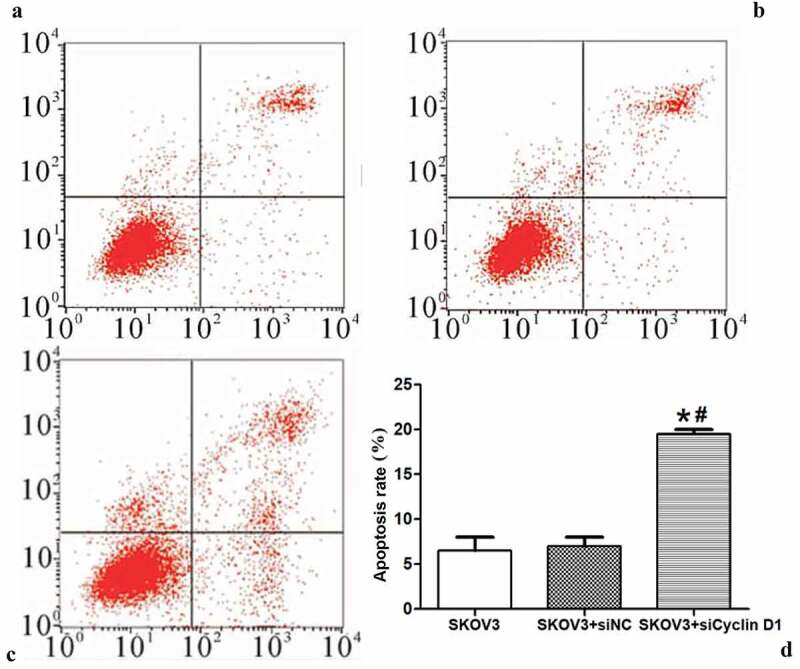
Effect of silencing CCND1 gene on apoptosis of ovarian cancer cells. A/B/C: SKOV3, SKOV3 + siNC and SKOV3 + siCCND1. D: Comparison of cell apoptosis rate among the three groups (*P < 0.01 *vs*. SKOV3 group, ^#^P < 0.05 *vs*. SKOV3 + siNC group)

The effect of silencing CCND1 gene on the apoptosis of ovarian cancer cells was detected by flow cytometry. The apoptosis rate of ovarian cancer cells was significantly higher in SKOV3 + siCCND1 group than those of SKOV3 and SKOV3 + siNC groups (P < 0.01) (Figure 7).

### Network diagram of CCND1-related proteins

**Figure 8.**
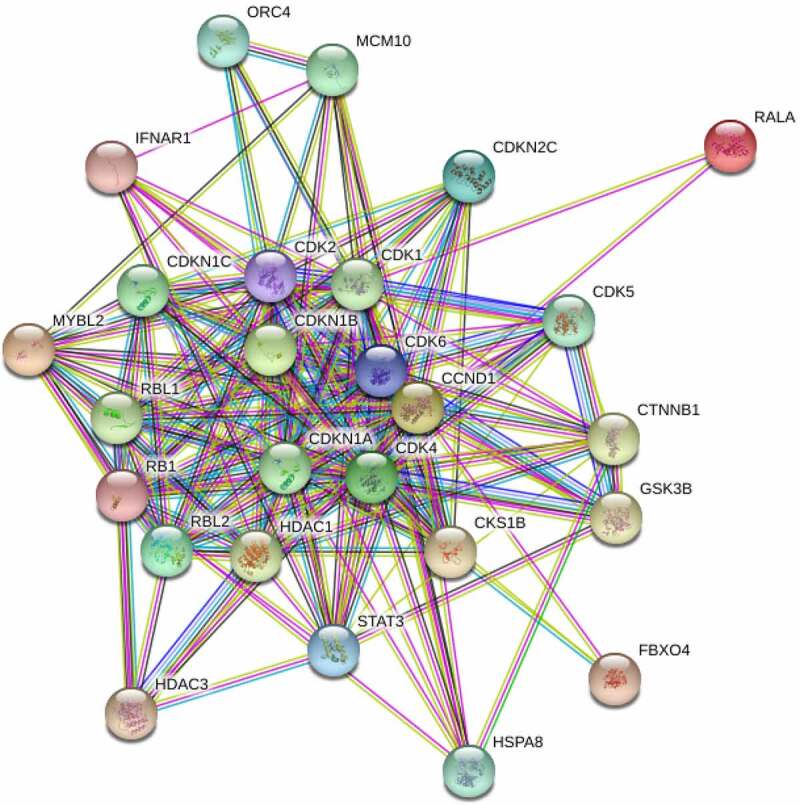
Network diagram of CCND1-related proteins

According to analysis based on the GeneCards, the major related proteins of CCND1 gene included RALA, MYBL2, CDKN1B, CDK4, RBL2, CDKN2C, STAT3, CDK2, RB1, IFNAR1, FBXO4, HDAC3, CKS1B, GSK3B, CTNNB1, HDAC1, RBL1, CDK1, CDKN1A, CDKN1C, MCM10, CDK5, HSPA8 and ORC4 (Figure 8). A network diagram of CCND1-related proteins was constructed using STRING. Enrichment analysis was conducted on the above related proteins which were mainly enriched in physiological processes such as regulation of cyclin-dependent protein serine/threonine kinase activity, G_1_/S transition in the mitotic cell cycle, negative regulation of transcription by RNA polymerase and reentry into mitotic cell cycle (Table 1).

**Table 1.** Enrichment process of CCND1 gene-related proteins

GO ID	Physiological process	Gene counting	P
GO:0000079	Regulation of cyclin-dependent protein serine/threonine kinase activity	14	1.30e-15
GO:0000082	G_1_/S transition in mitotic cell cycle	13	4.35e-12
GO:0000122	Negative regulation of transcription by RNA polymerase	7	1.02e-08
GO:0000320	Reentry into mitotic cell cycle	11	6.42e-05

## Discussion

Cervical cancer, endometrial cancer and ovarian cancer are the most common female malignancies. Its mortality rate ranks first among those of gynecological malignant tumors, and shows an upward trend annually. Ovarian cancer patients are typically diagnosed at an advanced stage due to the absence of symptoms, which may account for the low survival rates; the 10-year survival rate of patients diagnosed with advanced-stage ovarian cancer is 15%, compared to 55% for those diagnosed with the early-stage illness [13]. Patients with advanced ovarian cancer are usually treated by cytoreductive surgery combined with platinum therapy. However, the patients are prone to recurrence, distant metastasis and resistance to chemotherapeutic drugs, thereby having a poor prognosis [14,15]. Hence, exploring effective gene biomarkers of ovarian cancer is critical for understanding the mechanism of tumor formation, prognosis of patients and targeted drug therapy.

CCND1 is a cell cycle regulator. It is well-documented that many primary tumors are featured by overexpression of CCND1 gene, which is of great significance for the diagnosis and prognosis of tumors [16]. CCND1 gene (also known as CCND1, PRAD1 and BCL-1) is located on human chromosome 11q13, which encodes a protein consisting of 295 amino acid residues [17]. This gene is aberrantly highly expressed in many malignant tumors, and patients with high expression of CCND1 gene have a higher degree of malignancy and worse prognosis [18]. The Oncomine database, the world’s largest gene microarray database and data analysis and processing platform, involves many large-sample, high-quality gene microarrays and literature data, so it has high credibility and is helpful for exploring new cancer biomarkers and treatment targets [19]. In this study, after retrieving and analyzing the Oncomine database, it was found that CCND1 gene was differentially expressed in many tumors, such as bladder cancer and cervical cancer. A total of 57 studies indicated the differential expression of CCND1 gene, including 49 cases of high expression and 8 cases of low expression. By mining and analyzing 7 studies on the differential expression of CCND1 gene in ovarian cancer and normal ovarian tissue included in the Oncomine database, and by investigating the Adib Ovarian, Bonome Ovarian and Hendrix Ovarian microarrays, it was found that the expression of CCND1 gene was higher in ovarian cancer tissue than that in normal ovarian tissue.

The Kaplan-Meier Plotter database is a public database that contains the mRNA expression profile microarrays of 5 types of cancers, from which the correlation between gene expression and disease prognosis can be obtained. Shan *et al*. used the Kaplan-Meier Plotter database to study the correlation between the overexpression of CCND1 gene and prognosis of gastric cancer patients, and found that the overexpression of CCND1 gene shortened OS and PFS of patients [20]. In this study, the survival data of CCND1 gene were analyzed using the Kaplan-Meier Plotter database. The expression level of CCND1 gene significantly affected OS and PFS of ovarian cancer patients. PFS and OS of patients with high expression of CCND1 gene were markedly shortened, while the prognosis of patients with low expression of CCND1 gene may be improved. Thus, CCND1 gene may affect the prognosis of ovarian cancer patients, which is consistent with the results of previous studies. To further confirm the effect of CCND1 gene on ovarian cancer, the expression of CCND1 gene was compared between human ovarian cancer cell line SKOV3 and normal ovarian cells. The results demonstrated that the expression of CCND1 gene in SKOV3 cells was significantly higher than that in normal ovarian cells. Moreover, the influence of CCND1 gene on the cell cycle and apoptosis rate of ovarian cancer cells were further analyzed by silencing. After silencing of CCND1 gene, the percentage of cells in G_0_/G_1_ phase increased, the percentage of cells in S phase decreased, and the apoptosis rate reduced significantly. Hence, silencing CCND1 gene can effectively suppress the cell cycle and apoptosis of cancer cells, which is in line with the results of a previous literature [21].

The mechanism of CCND1 gene in ovarian cancer remains unclear. In this study, the related proteins of CCND1 gene were mined through the GeneCards database and included in enrichment analysis. They were mainly enriched in physiological processes such as regulation of cyclin-dependent protein serine/threonine kinase activity, G_1_/S transition in mitotic cell cycle, negative regulation of transcription by RNA polymerase and reentry into mitotic cell cycle. It has been reported that CCND1 gene can bind cyclin-dependent kinase 4 (CDK4) and CDK6 to form a complex, which cooperates with cyclin E-CDK2 complex to phosphorylate the serine and tyrosine residues of pRb and release transcription factor E2F, thus starting DNA synthesis, driving cells to transit from G_1_ phase to S phase, and leading to abnormal cell cycle and tumorigenesis [22].

## Conclusion

n summary, analysis based on the Oncomine database revealed that CCND1 gene was highly expressed in ovarian cancer tissue and associated with the prognosis of ovarian cancer patients. Preoperative evaluation of CCND1 gene expression in ovarian cancer patients may benefit the assessment of risk and prognosis, provide a new basis for CCND1 gene as a novel target for tumor therapy, and also pave the way for clinical treatment.

## Highlights

- CCND1 gene is highly expressed in ovarian cancer tissue.
- CCND1 gene is related to the prognosis of ovarian cancer patients.
- Preoperative evaluation of CCND1 expression is valuable for ovarian cancer patients.
